# Integrating highly active graphite nanosheets into microspheres for enhanced lithium storage properties of silicon†

**DOI:** 10.1039/d2ra06977f

**Published:** 2023-01-30

**Authors:** Yan Li, Dong Wang, Zhichao Liu, Xianzheng Liu, Jie Fu, Chunjie Zhang, Rui Zhang, Guangwu Wen

**Affiliations:** a School of Materials Science and Engineering, Shandong University of Technology Zibo 255000 P. R. China wangdong2@sdut.edu.cn wengw@sdut.edu.cn; b Shangdong Si-Nano Materials Technology Co., Ltd. Zibo 255000 P. R. China; c School of Materials Science and Engineering, Harbin Institute of Technology Harbin 150001 P. R. China

## Abstract

Integrating silicon (Si) and graphitic carbon into micron-sized composites by spray-drying holds great potential in developing advanced anodes for high-energy-density lithium-ion batteries (LIBs). However, common graphite particles as graphitic carbon are always too large in three-dimensional size, resulting in inhomogeneous hybridization with nanosized Si (NSi); in addition, the rate capability of graphite is poor owing to sluggish intercalation kinetics. Herein, we integrated graphite nanosheets (GNs) with NSi to prepare porous NSi-GN-C microspheres by spray-drying and subsequent calcination with the assistance of glucose. Two-dimensional GNs with average thickness of ∼80 nm demonstrate superior lithium storage capacity, high conductivity, and flexibility, which could improve the electronic transfer kinetics and structural stability. Moreover, the porous structure buffers the volume expansion of Si during the lithiation process. The obtained NSi-GN-C microspheres manifest excellent electrochemical performance, including high initial coulombic efficiency of 85.9%, excellent rate capability of 94.4% capacity retention after 50 repeated high-rate tests, and good cyclic performance for 500 cycles at 1.0 A g^−1^. Kinetic analysis and *in situ* impedance spectra reveal dominant pseudocapacitive behavior with rapid and stable Li^+^ insertion/extraction processes. *Ex situ* morphology characterization demonstrates the ultra-stable integrated structure of the NSi-GN-C. The highly active GN demonstrates great potential to improve the lithium storage properties of Si, which provides new opportunity for constructing high-performance anodes for LIBs.

## Abstract 2

Highly active graphite nanosheets are integrated with Si nanoparticles to prepare porous microspheres by spray-drying and a subsequent annealing process, which demonstrate superior lithium storage properties.

## Graphical abstract

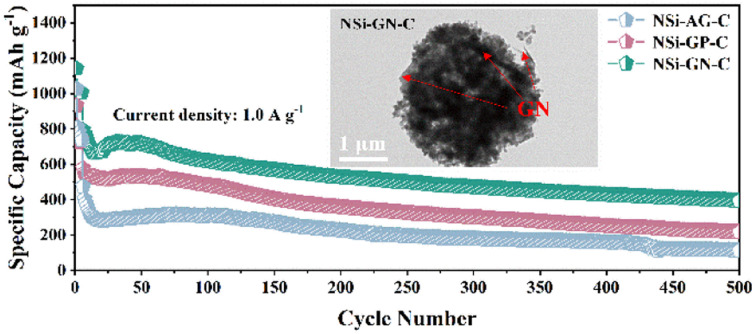

## 1. Introduction

With the rapid development of electrical vehicles and portable electronic devices, higher requirements are put forward for the energy density and cyclic life of lithium-ion batteries (LIBs).^1–3^ Currently, graphite is widely used as anode material due to its high electrical conductivity, good reversibility, and low cost.^4,5^ However, the commonly used graphite anode suffers from low theoretical specific capacity (372 mA h g^−1^), which dramatically limits the application potential of LIBs.^6–8^ Among the numerous anode materials, silicon (Si) has emerged as the most promising substitute for commercial graphite anodes owing to its low lithiation/delithiation potential, unparalleled theoretical specific capacity (4200 mA h g^−1^), and environmental friendliness.^9,10^ However, the practical application of Si-based anodes is limited owing to its low electrical conductivity.^11^ Moreover, Si also suffers from large volume changes (300–400%) during lithiation/delithiation processes, leading to serious pulverization, contact loss of electrode (active materials, current collectors, and conductive additives), and continuous growth of a solid electrolyte interface (SEI) layer during the charge/discharge processes, thereby reducing the cyclic stability and rate capability.^12–15^

The above problems with Si could be addressed by means of nanostructure engineering and hybridization with carbon. Notably, nanostructure can alleviate the absolute volumetric change of Si and have considerably improved the cyclability.^2,16^ However, downsizing Si particles alone cannot conquer the issue of low conductivity, and also cause more electrolyte consumption to form SEI film, thus leading to low initial coulombic efficiency (ICE).^6,17^ In addition, Si nanoparticles are easy to agglomerate, leading to much lower volume density than their microsized counterparts, which is detrimental to practical applications of Si anodes.^18,19^ Hybridization with carbon is another effective way to enhance the electrochemical performance of Si anodes.^20,21^ Highly conductive carbon could effectively improve the overall electrons transfer kinetics of composites. Moreover, the intrinsic flexibility of carbon matrix could further buffer the volume expansion and shrinkage of Si nanoparticles during lithiation/delithiation.^22–25^ On the basis of above mentioned strategies, constructing Si/carbon composites with micron size demonstrate great potentials on improving the electrochemical performance of Si-based anode.

Spray-drying is a facile and common method to prepare micron-sized particles owing to the advantages of large-scale production, easy operating, and environmental friendliness.^26,27^ Various kinds of Si/carbon microparticles with different morphology and composition have been successfully synthesized by spray-drying method combined with subsequent annealing treatment. Thereinto, graphitic carbon materials, such as graphite and graphene, were often needed in these composites, acting as conductive agent to improve the whole electronic conductivity.^28^ For example, Zhang *et al.* proposed the direct growth of amorphous Si/C layers on graphitized carbon black particles distributed in microspheres by spray-drying method.^29^ Peng *et al.* prepared a dual-shell structure Si@SiO_*x*_@graphite/graphene (SGGr) by spray-drying.^30^ It can be seen that graphite is common conductive agent for spray-drying induced synthesis of Si/carbon composites. However, conventional graphite particles are too large in size, always resulting in uneven dispersion with Si nanoparticles. Moreover, graphite anode suffers from unsatisfactory rate capabilities due to sluggish intercalation/deintercalation kinetics. Therefore, delicate compositional design of micronsized Si/carbon composites using spray-drying methods is highly desired.

In this work, we use highly active graphite nanosheets (GNs) as conductive agent, and synthesize the nanosilicon-GN-glucose microspheres by spray-drying, which were further carbonized into porous nanosilicon-GN-carbon (NSi-GN-C) microspheres under high temperature. GN demonstrates lamellar structure and much superior rate capabilities to the graphite powder (GP) and artificial graphite (AG). In the obtained NSi-GN-C, NSi and GNs assemble into integrated spherical configuration with the adhesive effect of glucose-derived carbon. Thereinto, the highly active GN inserts and/or coats in the microspheres, acting as both conductive agent and structural stabilizer to improve the lithium storage properties, while glucose-derived carbon coats on the surface of Si, further accelerating electrons transfer. In addition, the synthesized NSi-GN-C microspheres possess hierarchical internal pores, which provide enough space to relieve huge volume expansion of Si and accelerate the Li^+^ diffusion kinetics in the electrode. When evaluated as LIBs anodes, the NSi-GN-C microspheres manifest much superior lithium storage properties to the NSi and NSi-C, demonstrating a high initial coulombic efficiency (ICE) of 85.9%, good rate capability (94.4% capacity retention after cycles at 50 times high-rate), and excellent cyclic performance (high reversible capacity of 400 mA h g^−1^ after 500 cycles at 1.0 A g^−1^). Kinetics analysis demonstrates that the lithium storage behavior of the NSi-GN-C electrode is dominantly controlled by surface pesudocapacitive process, reaching a high capacitive contribution of 96.8%. Moreover, the NSi-GN-C electrode exhibits an ultra-low electrode swelling of 3.8%, much lower than that of NSi (28.8%). In addition, the NSi-GN-C microspheres also show superior electrochemical performance to the NSi-GP-C and NSi-AG-C electrodes, again confirming the great potentials of highly active GN as an advantageous component for Si anode.

## 2. Experimental

### 2.1. Preparation of NSi-GN-C microspheres

NSi was obtained from Shanghai Chaowei Nano Co. 2.0 g of glucose was dispersed in 50 mL of deionized water and stirred for 15 min. 1.0 g of NSi powder and 1.0 g of GN were added into the glucose solution in turn, stirred and sonicated for 1 h to obtain a homogeneous suspension. The annealing precursor was generated from the suspension by spray-drying under the following conditions: the suspension delivery rate was 15 mL min^−1^; the inlet temperature of the spray dryer was 200 °C. The obtained annealing precursors obtained by spray-drying were denoted as NSi-GN-FC. Finally, the NSi-GN-FC was heated at 800 °C for 3 h with a ramping rate of 5 °C min^−1^ to obtain the Si/carbon composite, which was denoted as NSi-GN-C. In addition, by replacing GN with graphite powder (GP) or artificial graphite (AG), the NSi-GP-C or NSi-AG-C were prepared under the same synthetic conditions.

For comparison, under the same experimental conditions, the mass ratios of NSi to GN were adjusted to 0.5 : 1 and 1.5 : 1 to obtain the NSi-GN-C-0.5 and NSi-GN-C-1.5 composites. In addition, we also prepared NSi-C without adding GN.

### 2.2. Characterization

The microstructure and morphology of the samples were tested by transmission electron microscopy (TEM, JWJGS-032, 200 kV) and scanning electron microscopy (SEM, quanta 250) with energy dispersive X-ray spectroscopy. The chemical composition was obtained by X-ray diffraction (XRD-6000, Cu Kα, 30.0 kV, 20.0 mA) and Raman spectroscopy (Horiba Scientific LabRAM HR Evolution). The carbon content was recorded by thermogravimetric analysis (TGA, DTG-60H). The Brunauer–Emmett–Teller (BET) surface area and pore structure were characterized by N_2_ adsorption/desorption isotherms using ASAP 2020 (Micromeritics) at 77 K.

### 2.3. Electrochemical measurement

The working electrode was prepared by casting a slurry consisting of active material, Super-P, and sodium alginate in a weight ratio of 8 : 1 : 1 onto a copper foil. The mass loading of active material for each electrode was about 1.5 mg cm^−2^. The electrolyte as 1.0 M LiPF_6_ in ethylene carbonate (EC)/diethyl carbonate (DEC) (1 : 1 by volume) with 5.0 vol% fluoroethylene carbonate (FEC) and 1.0 vol% vinyl carbonate (VC). The 2025 type button cell was assembled with Celgard 2325 film and lithium metal foil as separator and counter/reference electrodes, respectively, to evaluate the electrochemical performance of the composites. Galvanostatic charge/discharge experiments were performed using a multichannel battery testing system (LAND) between 0.01 and 2.0 V. Cyclic voltammetry (CV) was performed over a potential range of 0.01–2.0 V at a scan rate of 0.1 mV s^−1^ by a CHI660A electrochemical workstation. Electrochemical impedance spectroscopy (EIS) was performed in the frequency range of 100 kHz and 0.01 Hz.

## 3. Results and discussion

**Fig. 1:**
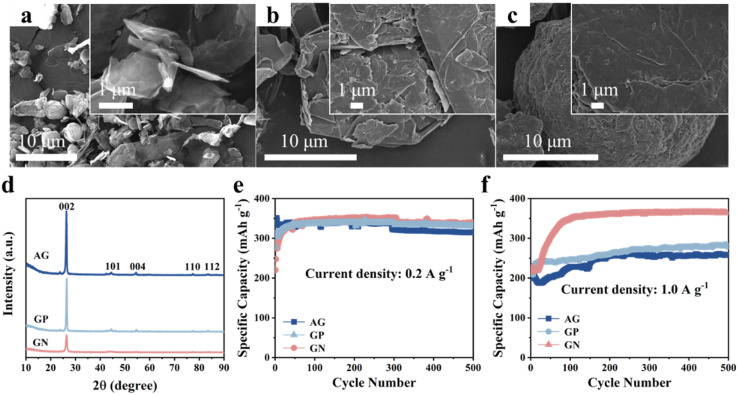
Characterization and lithium storage properties of the GN, GP, AG. SEM images of (a) GN, (b) GP, (c) AG. (d) XRD patterns of GN, GP, AG. Cyclic performance of the AG, GP, GN electrodes at (e) 0.2 A g^−1^ and (f) 1.0 A g^−1^.

First, we compared the microstructure and electrochemical performance of the three graphitic carbon, that is, AG, GP, GN. It can be clearly seen that the GN presents angular nanosheet structure with an average thickness of ∼80 nm (Fig. 1a). Two-dimensional (2D) nanosheets with large specific surface area could provide more electrochemically active sites and shorten ions diffusion path, leading to good lithium storage properties. Moreover, 2D morphology of GN is beneficial to connect and cover Si nanoparticles.

In comparison, the GP shows irregular shape with particle size of as large as 30 μm (Fig. 1b). In addition, the AG presents elliptical-like morphology in shape, and the particle sizes show a wide range from 15 to 25 μm (Fig. 1c). Fig. S1† shows the Raman spectra of three kinds of graphitic carbon, and the *I*_D_/*I*_G_ of AG, GN and GP are 1.3, 0.94 and 0.86, respectively. XRD patterns of the AG, GN, GP are shown inFig. 1d, corresponding to the standard card of graphite (PDF# 41-1487). Cyclic performances of these three graphitic carbons are measured at various current densities (Fig. 1e and f). It can be seen that these three graphitic carbon electrodes all manifest excellent cyclic stability at low current density of 0.2 A g^−1^ and high current density of 1.0 A g^−1^. Moreover, although the GN electrode shows similar specific capacity with the GP and AG at 0.2 A g^−1^, it delivers much higher specific capacity at 1.0 A g^−1^, reaching 363 mA h g^−1^ after 500 cycles. This should be ascribed to the 2D nanosheet structure with more active sites and shortened Li^+^ diffusion path, endowing the GN with fast electrochemical reaction kinetics.

**Fig. 2:**
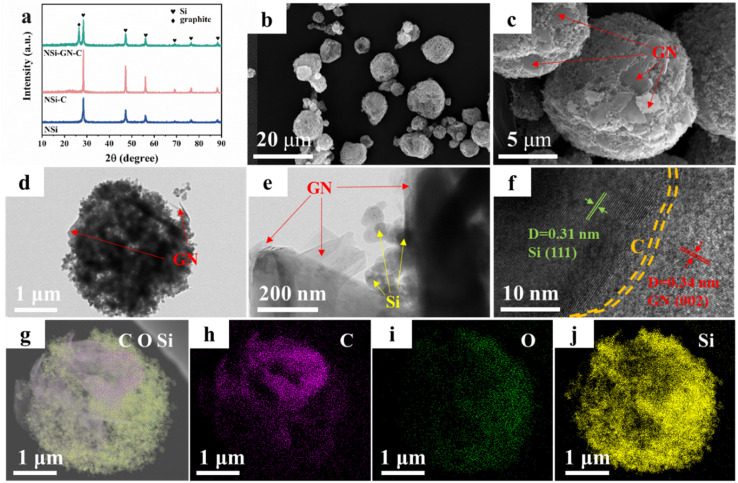
Characterization of the NSi-GN-C microspheres. (a) XRD patterns of the NSi, NSi-C, NSi-GN-C. (b and c) SEM images of the NSi-GN-C microspheres. (d and e) TEM images of the NSi-GN-C microspheres. (f) HRTEM image of the NSi-GN-C microspheres. (g–j) Elemental mapping of Si, C and O elements for the NSi-GN-C microspheres.

On the basis of the above results, the highly active GN was selected as the graphitic carbon for preparation of the NSi-GN-C composites by spray-drying and subsequent annealing process. XRD patterns of the NSi (raw materials), NSi-C, and NSi-GN-C composites are displayed in Fig. 2a, showing that the diffraction peaks of NSi-GN-C combine those of NSi and GN without other impurities. In addition, the small broad peaks at ∼23° of the NSi-C and NSi-GN-C should be assigned to glucose-derived amorphous carbon. Fig. S2† shows that the raw material NSi possesses an average particle size of ∼50 nm. It can be seen that the obtained-NSi-GN-C displays typical spherical morphology with micrometer scale (Fig. 2b and c), in which the Si nanoparticles tightly bind with the GN under the adhesive effect of glucose-derived carbon. Moreover, the surface of NSi-GN-C microspheres is covered by 2D GN. TEM images indicate that except coating on the surface, the GN are also inserted into the inside of the microspheres. Note that even after drastic ultrasonic treatment during TEM sample preparation, the composites still maintain integrated spherical morphology, in which Si nanoparticles are uniformly distributed with GN (Fig. 2d and e). High-resolution TEM (HRTEM) image shows distinct lattice fringes with interplanar spacings of 0.31 and 0.34 nm, assigned to (111) plane of Si and (002) plane of GN, respectively (Fig. 2f). Moreover, it could also be seen that the surface of NSi is uniformly coated by amorphous carbon layer derived from glucose. In addition, the high-angle annular dark-field scanning TEM (HAADF-STEM) and elemental mapping images demonstrate the mutual distribution of C, O and Si elements in the NSi-GN-C microspheres, further confirming the successful synthesis of Si/carbon composites (Fig. 2g–j). The dispersed GN along with glucose-derived carbon could effectively enhance the electronic conductivity of composites, protect the NSi from severe interface side reactions, and further buffer the volume variation during repeated lithiation/delithiation processes. For comparison, we also prepared the NSi-GN-C-0.5 and NSi-GN-C-1.5 composites with different contents of Si. It can be seen that the NSi-GN-C-0.5 composites show discrete structure (Fig. S3a and b†), while the NSi-GN-C-1.5 composites display spherical-like shapes (Fig. S3c and d†).

**Fig. 3:**
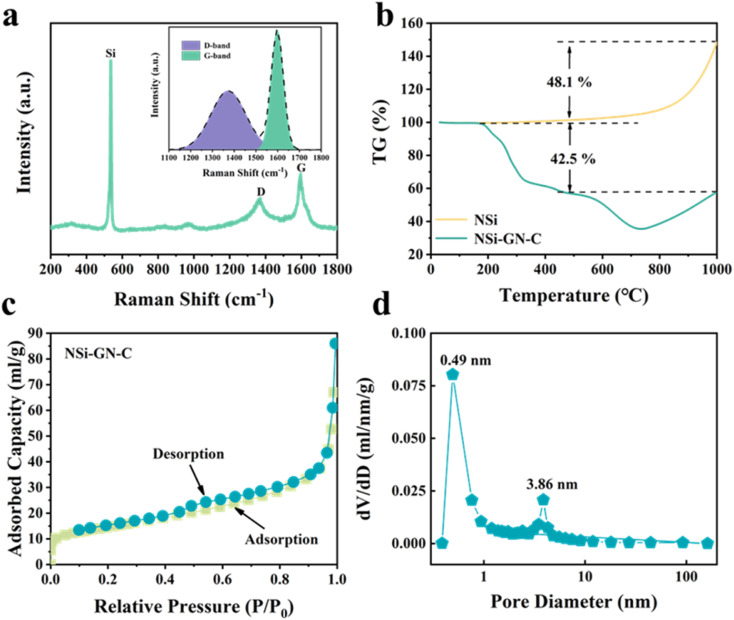
Characterization of the NSi-GN-C microspheres. (a) Raman spectrum of the NSi-GN-C microspheres. (b) TGA curves of the NSi and NSi-GN-C microspheres. (c) Nitrogen adsorption/desorption isotherms and (d) pore size distribution plot of the NSi-GN-C microspheres.

Fig. 3a and S4† show the Raman spectrum of these three NSi-GN-C samples. The peaks at about 291.2, 511.2, and 931.1 cm^−1^ belong to the vibrational bands of crystalline Si, as also observed in Fig. S5.† The other two broad peaks at about 1344.3 and 1592.8 cm^−1^ are assigned to the defect-induced D band and in-plane vibration G band of carbonaceous materials, again indicating the successful composition of silicon and carbon. The *I*_D_/*I*_G_ value of the NSi-GN-C is 1.01, in the range from 0.94 (*I*_D_/*I*_G_ value of the GN) to 1.23 (*I*_D_/*I*_G_ value of the glucose-derived carbon) (Fig. S6†). Furthermore, the carbon content in these composites is calculated based on TGA measurement in air atmosphere (Fig. 3b and S7†). The weight loss (42.5%) of NSi-GN-C microspheres from 200 °C to 1000 °C should be due to the thermal decomposition of GN and pyrolytic carbon, and the oxidation of Si nanoparticles in air. Note that the Si oxidation-induced weight increasement is ∼48.1%.^31–33^ Therefore, the carbon contents are calculated to be 42.5 wt%, 75.7 wt%, and 37.6 wt% for the NSi-GN-C, NSi-GN-C-0.5, and NSi-GN-C-1.5, respectively. N_2_ adsorption/desorption isotherms of the NSi-GN-C microspheres demonstrate type-IV isotherms, while the hysteresis loops in high-middle pressure indicates the existence of mesopores, and the rise in low pressure is due to the micropores. Corresponding pore size distribution plot further confirms the hierarchically porous structure, of which two intensive pores centering at 0.49 and 3.86 nm are observed, consistent with the isotherms result. The pore structure should come from the assembly process of GN and NSi, as well as the carbonization of glucose. The Brunauer–Emmett–Teller (BET) surface areas of the NSi-GN-C sample is 50.6 m^2^ g^−1^, lower than that of the NSi (254.4 m^2^ g^−1^) (Fig. S8†). The NSi-GN-C-0.5 and NSi-GN-C-1.5 samples also show hierarchically porous structure (Fig. S9†). Mesopores could promote electrolyte infiltration and accelerate Li ions transfer, while micropores could act as extra adsorption sites to improve lithium storage capacity.

**Fig. 4:**
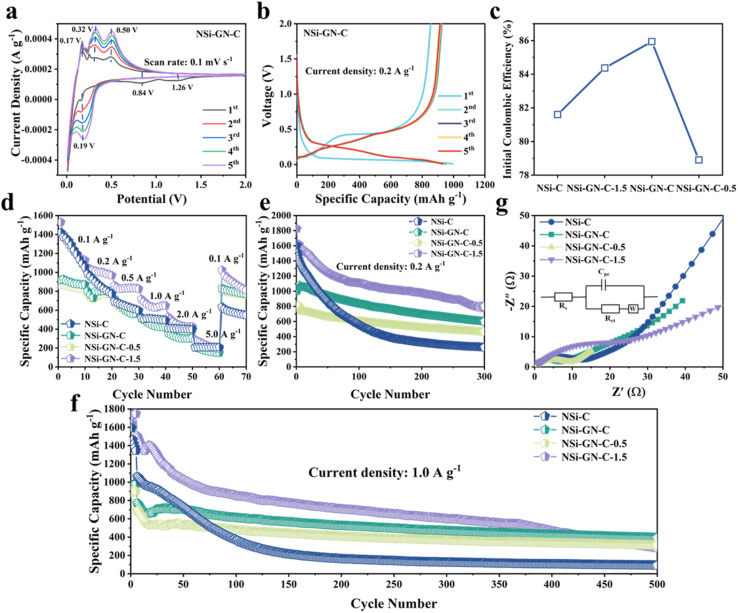
Lithium storage properties. (a) CV curves of the NSi-GN-C microspheres. (b) Charge/discharge curves of the NSi-GN-C microspheres at 0.2 A g^−1^ from 0.01 to 2 V. (c) Comparison of ICE of the NSi-C, NSi-GN-C, NSi-GN-C-0.5, NSi-GN-C-1.5. (d) Rate capabilities of the NSi-C, NSi-GN-C, NSi-GN-C-0.5, NSi-GN-C-1.5 at different current densities. Cyclic performance of the NSi-C, NSi-GN-C, NSi-GN-C-0.5, NSi-GN-C-1.5 at (e) 0.2 A g^−1^ and (f) 1.0 A g^−1^. (g) Nyquist plots of the NSi-C, NSi-GN-C, NSi-GN-C-0.5, NSi-GN-C-1.5 after 150 cycles. Inset shows the equivalent circuit.

Then, the lithium storage properties of the NSi-GN-C were evaluated using half cells in the voltage range between 0.01 and 2.0 V. CV of the NSi-GN-C electrode was performed at a scan rate of 0.1 mV s^−1^ (Fig. 4a). In the first lithiation process, owing to the generation of the SEI film, two broad cathodic peaks occur at ∼1.26 V and ∼0.84 V.^34^ A strong cathodic peak (∼0.19 V) should be attributed to the intercalation of Li^+^ ions into Si to obtain Li_*x*_Si.^2,33,35^ Anodic peaks (0.17 V, 0.32 V and 0.50 V) correspond to gradual deintercalation of Li_*x*_Si to generate amorphous Si.^36,37^ CV curves of NSi-GN-C-0.5 and NSi-GN-C-1.5 are also shown in Fig. S10.†

As shown in Fig. 4b, the NSi-GN-C electrode delivers initial discharge/charge capacities of 993.8 and 854.0 mA h g^−1^ at 0.2 A g^−1^, achieving a high ICE of 85.9%. The capacity loss in the first cycle should be due to the irreversible formation of SEI layer.^38^ In the subsequent cycles, the charge/discharge profiles overlap well with each other, indicating excellent cyclic stability of the NSi-GN-C, mainly owing to the synergistic effect of highly conductive GN and continuous amorphous carbon matrix. Note that the ICE of the NSi-GN-C is larger than those of the NSi-GN-C-0.5 (78.9%) and NSi-GN-C-1.5 (84.3%) (Fig. 4c). Rate capabilities of the NSi-C, NSi-GN-C, NSi-GN-C-0.5, and NSi-GN-C-1.5 electrodes are illustrated in Fig. 4d. Although the reversible specific capacity of the NSi-GN-C electrode (880 mA h g^−1^ at 0.1 A g^−1^) is lower than those of NSi-C (1248 mA h g^−1^) and NSi-GN-C-1.5 (1264 mA h g^−1^), the capacity retention of the NSi-GN-C as the current density was restored to 0.1 A g^−1^ after 50 times high-rate test (5.0 A g^−1^) is 94.4%, much higher than those of NSi-C (46.0%) and NSi-GN-C-1.5 (70.0%). The co-interaction of GN with amorphous carbon promotes rapid electron transfer and thus enhances the electrical conductivity of the whole electrode. Fig. 4e and f show the cyclic properties of the composite at 0.2 and 1.0 A g^−1^, respectively. The NSi-GN-C microspheres could maintain a reversible specific capacity of 599.4 mA h g^−1^ after 300 cycles at 0.2 A g^−1^, which is larger than that of the NSi-GN-C-0.5 (463.3 mA h g^−1^), mainly due to smaller Si content of NSi-GN-C-0.5. In addition, although the NSi-C and NSi-GN-C-1.5 electrodes deliver larger specific capacities than the NSi-GN-C in the initial cycles, they show much lower capacity retention of 16% (NSi-C), 43.7% (NSi-GN-C-1.5) than the NSi-GN-C (60%). Furthermore, an elevated current density of 1.0 A g^−1^ was further applied to investigate the long-term cyclic performance (Fig. 4f). It can be seen that the NSi-GN-C electrode demonstrates excellent cyclic stability for 500 cycles, delivering a considerable reversible capacity of 400 mA h g^−1^ with a quite slow capacity reduction rate of 0.08% per cycle. For comparison, the NSi-GN-C-0.5 and NSi-GN-C-1.5 electrodes show worse cyclic stability and lower capacities of 312.9 mA h g^−1^ and 284.5 mA h g^−1^ after 500 cycles. The excellent cyclic performance of the NSi-GN-C is mainly due to the integrated micronsized structure, of which the highly active GN and amorphous carbon promotes the rapid electrons transfer and hinder the large volume changes during lithiation/delithiation processes. In addition, the interior pore structure could further provide buffer space for accommodating the volume expansion. Fig. 4g shows the Nyquist plots and corresponding equivalent circuit of the NSi-C, NSi-GN-C, NSi-GN-C-0.5 and NSi-GN-C-1.5 electrodes after 150 cycles. The *R*_s_ represents the cell components (electrolyte, electrode, diaphragm), while the charge transfer resistance is represented by *R*_ct_, and *C*_pe_ represents the bilayer capacitance. It can be seen that the NSi-GN-C electrode shows a much smaller semicircle than the NSi-C and NSi-GN-C-1.5 electrodes, indicating better electronic conductivity endowed by the GN component.

**Fig. 5:**
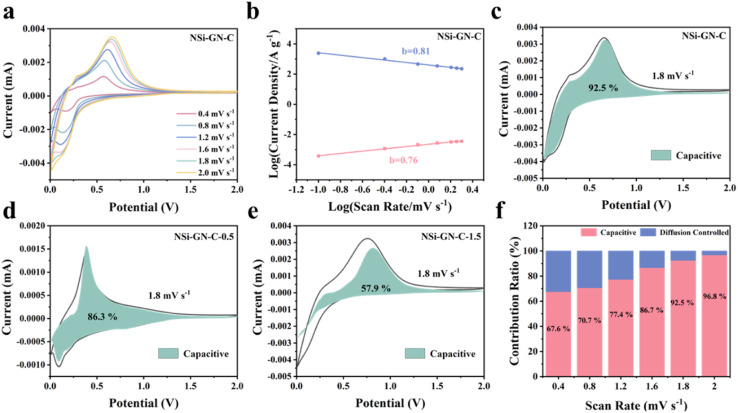
Kinetic analysis. (a) CV curves of NSi-GN-C microspheres electrode at different scan rates. (b) Relationship between peak current and scan rate. Capacitive contribution at 1.8 mV s^−1^ of (c) the NSi-GN-C microspheres, (d) NSi-GN-C-0.5, (e) NSi-GN-C-1.5. (f) Ratio of capacitive and diffusion-controlled contributions at different scan rates of the NSi-GN-C microspheres.

Furthermore, we investigated the kinetics behavior of these NSi-GN-C electrodes. Fig. 5a shows the CV curves of the NSi-GN-C electrode, of which the peaks maintain pristine shape with stepwise increasing the scan rates from 0.4 to 2.0 mV s^−1^. Similarly, the CV curves of NSi-GN-C-0.5 and NSi-GN-C-1.5 electrodes did not change (Fig. S12a and d†). The relationship between the peak current (*i*) and the scan rate (*v*) can indirectly illustrate the kinetics control process.^39^ It could be investigated based on the following formulas:

1 *i* = *av*^*b*^

2 log(*i*) = *b* log(*v*) + log(*a*)

The value of *b* is between 0.5 and 1.0, which is determined from the slope of the log *i versus* log *v* plot. As we all know, for diffusion control process, *b* value is close to 0.5; for surface control capacitance behavior, *b* value is close to 1.^40,41^ The calculated *b* values of the NSi-GN-C electrode are 0.81 and 0.76 for cathodic and anodic processes, respectively (Fig. 5b), indicating a more favored surface-induced capacitance process of NSi-GN-C. On the contrast, the *b* values for the NSi-GN-C-0.5 and NSi-GN-C-1.5 electrodes are lower, that is, 0.79/0.58 and 0.44/0.49, respectively (Fig. S12b and e†). Moreover, the capacitive contribution of the NSi-GN-C electrode were further quantitatively extracted based on simulating the current response *i*(V), according to the following equations in which both *k*_1_ and *k*_2_ are constant values, *k*_1_*v* and *k*_2_*v*^1/2^ stand for the capacitive-controlled and diffusion-dominated processes, respectively.^42,43^

3 *i*(V) = *k*_1_*v* + *k*_2_*v*^1/2^

As shown in Fig. 5c–e, the NSi-GN-C, NSi-GN-C-0.5 and NSi-GN-C-1.5 electrodes show dominant capacitance contributions of 92.5%, 86.3% and 57.9% (cyan region) at 1.8 mV s^−1^, respectively. Furthermore, the ratios of the capacitive contribution in the total capacity increase gradually along with increased scan rates (Fig. 5f, S12c and f†). Importantly, the capacitive contribution of the NSi-GN-C electrode at each scan rate is significantly higher than that of the NSi-GN-C-0.5 and NSi-GN-C-1.5 electrodes. These results suggest that the NSi-GN-C microsphere with porous structure and appropriate content of GN can accelerate electrons transfer and ions diffusion, leading to rapid electrochemical reaction kinetics.

**Fig. 6:**
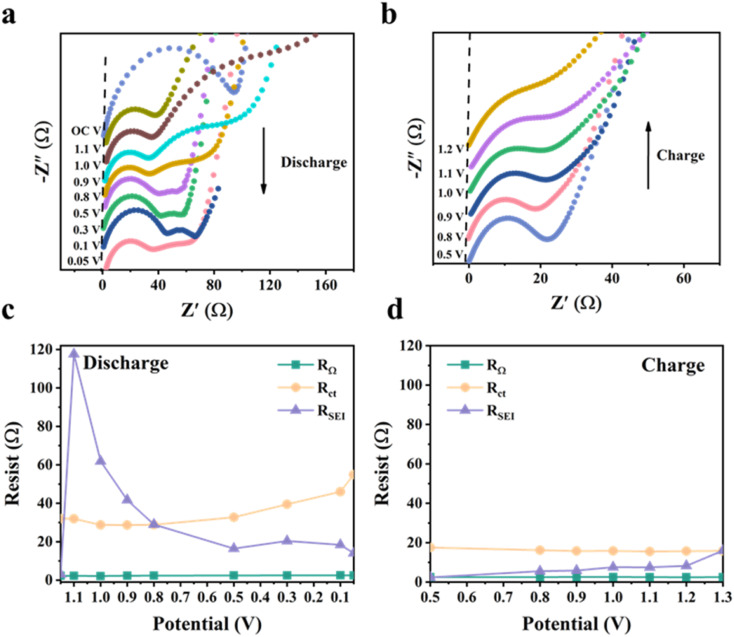
*In situ* EIS characterization of the NSi-GN-C. (a and b) Nyquist plots of NSi-GN-C microspheres at different potentials during GCD processes. (c and d) Corresponding impedances at different potentials during the GCD processes.

We further performed *in situ* EIS analysis of the NSi-GN-C microspheres to investigate the electrochemical behavior (Fig. 6). The circuit diagram of impedance fitting is shown in Fig. S13.† During the first discharge process, the SEI resistance (*R*_SEI_) increases from 3.1 Ω (open circuit potential) to 117.6 Ω (at 1.1 V), followed to a very low resistance (14.1 Ω) at 0.5 V, after which it remains almost constant (Fig. 6a and c). The former increasement is attributed to the formation of loose and resistive SEI film resulting from the electrolyte decomposition and other irreversible side reactions.^44,45^ Then, stable SEI layer is gradually built up when potential reaches to about 0.5 V. From the discharge process to charge process (Fig. 6b and d), the *R*_SEI_ first decreases and then gradually increases. This is probably related to slight structure changes in electrode structure upon cycling.^44^ Moreover, the *R*_ct_ keeps almost unchanged with slight increase during discharging process, indicating stable charge transfer kinetics during lithiation and delithiation processes. In addition, the *R*_ct_ values during discharge process are larger than those of charge, which means that the lithiation has less diffusivity of lithium ions within NSi-GN-C electrode in comparison with the delithiation.^46^ As for the intrinsic ohmic resistances (*R*_Ω_, represents the total resistances of electrolyte, electrode, and separator), it does not change significantly during the entire test.^47^ These features indicate the formation of robust SEI layer, as well as the stable Li^+^ insertion/extraction kinetics.

**Fig. 7:**
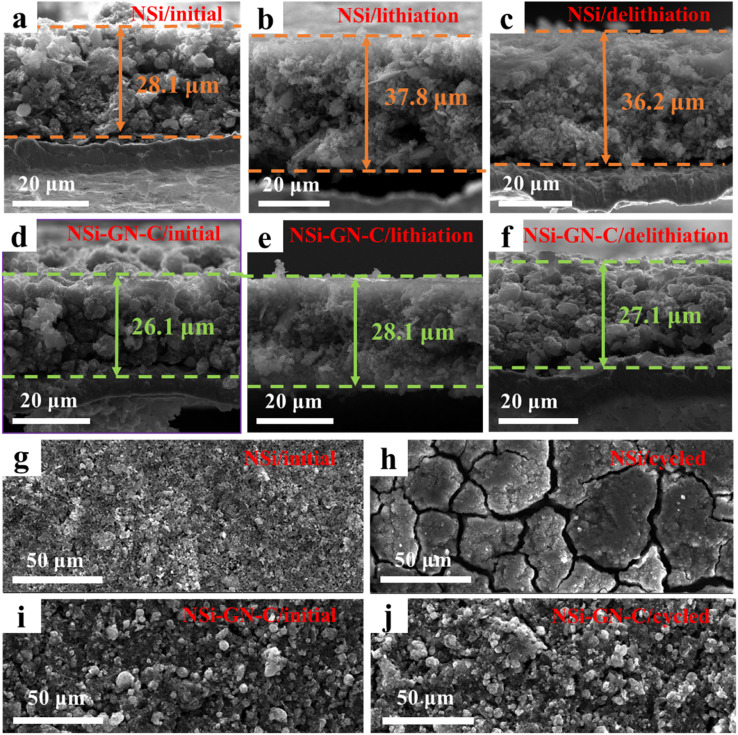
*Ex situ* microstructure characterization. Cross-sectional SEM images of the NSi electrode films (a) before cycling, (b) after lithiation, and (c) after delithiation. Cross-sectional SEM images of the NSi-GN-C electrode films (d) before cycling, (e) after lithiation, and (f) after delithiation. Top-view SEM images of the NSi and NSi-GN-C (g, i) before and (h, j) after 200 cycles.

To further study the structural stability of the NSi-GN-C microspheres, cross-sectional and top-view morphology evolutions of the NSi and NSi-GN-C electrodes before and after cycling were obtained. As shown in Fig. 7a–c, the initial NSi electrode shows an average film thickness of 28.1 μm, while the film thicknesses after first lithiation and first delithiation processes are 37.8 and 36.2 μm, respectively, indicating the electrode film expansion of 28.8%. By contrast, the NSi-GN-C electrode manifest much smaller film expansion of 3.8% (Fig. 7d–f), demonstrating that the GN and glucose-derived carbon, as well as the rich porosity of the microspheres could effectively accommodate the large volume variation of Si and improve the structural stability. Low electrode film expansion is critical for the practical application of Si-based anodes. Fig. 7g–j depicts the top-view microstructural evolution after 200 cycles. It can be clearly seen that both the NSi and NSi-GN-C electrodes show flat surface before cycling. However, the surface microstructure of the NSi electrode is severely damaged with large cracks after 200 cycles (Fig. 7g and h), mainly due to the large volume expansion of Si and agglomeration of nanoparticles during repeated charge/discharge processes. On the other hand, the NSi-GN-C electrode shows almost unchanged surface with only little cracks (Fig. 7i and j). Fig. S14† further indicates that even after repeated charging/discharging cycles, GN is still tightly coated/inserted in the microspheres. The stable electrode structure is beneficial for long-term cyclic performance.

**Fig. 8:**
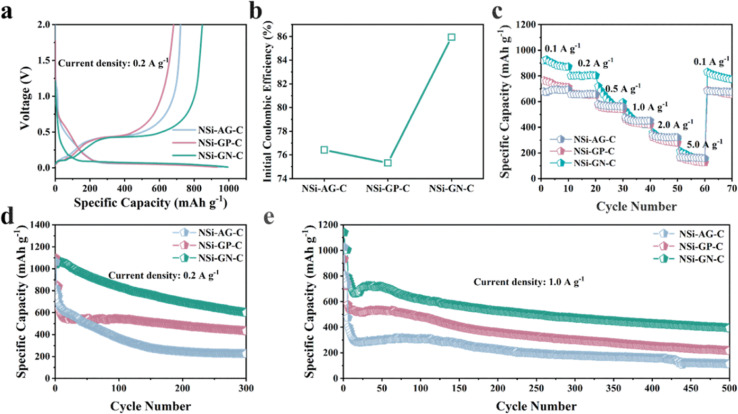
Lithium storage properties of the NSi-AG-C, NSi-GP-C, and NSi-GN-C. (a) Charge/discharge curves of the NSi-AG-C, NSi-GP-C, and NSi-GN-C of the first cycle (b) Comparison of the ICE. (c) Rate capabilities of the NSi-AG-C, NSi-GP-C, and NSi-GN-C at different current densities. Cyclic performance of the NSi-AG-C, NSi-GP-C and NSi-GN-C at (d) 0.2 A g^−1^ and (e) 1.0 A g^−1^.

Furthermore, to confirm the structural advantages of highly active GN in our work, we synthesized the NSi-AG-C and NSi-GP-C composites under the same conditions as that of the NSi-GN-C. The lithium storage properties of these three composites were compared using half-cells in the voltage range of 0.01 to 2.0 V. The first charge/discharge curves of these three electrodes are depicted in Fig. 8a, which show almost identical profile shapes, indicating similar lithiation/delithiation behaviors. Moreover, the NSi-GN-C electrode delivers larger reversible capacity than the other two electrodes. Fig. 8b further compares the ICE, showing that the NSi-GN-C electrode manifests much higher ICE than the NSi-AG-C (76.5%) and NSi-GP-C (75.3%). Rate capabilities of the NSi-AG-C, NSi-GP-C and NSi-GN-C electrodes are shown in Fig. 8c. After high-rate measurement at 5.0 A g^−1^, the recovered specific capacity of NSi-GN-C electrode at 0.1 A g^−1^ is 854.0 mA h g^−1^, higher than those of NSi-AG-C electrode (722.3 mA h g^−1^) and NSi-GP-C electrode (682.0 mA h g^−1^). Cyclic performance was also compared at 0.2 and 1.0 A g^−1^ (Fig. 8d and e). The specific capacity of NSi-GP-C composite is 431.7 mA h g^−1^ after 300 cycles at 0.2 A g^−1^, while the NSi-AG-C composite is only 224.2 mA h g^−1^. Compared to the other two composites, the NSi-GN-C electrode still delivers a higher capacity of 599.4 mA h g^−1^. In addition, the NSi-GN-C electrode also show the highest specific capacity compared with the other two electrodes after 500 cycles at 1.0 A g^−1^. The above results further confirm that the GN is advantageous conductive agent and structure stabilizer for the Si/carbon composites.

## 4. Conclusions

In summary, integrated NSi-GN-C microspheres with hierarchical pores were synthesized by spray-drying and subsequent high-temperature calcination, using GN and glucose as conductive agent, and adhesive as well as precursor of carbon, respectively. Owing to the highly conductive and active GN, integrated microspherical morphology, and porous structure, the NSi-GN-C microspheres show superior lithium storage properties than the NSi-GP-C and NSi-AG-C counterparts, in view of high ICE of 85.9%, excellent rate capability, as well as good cyclic performance (599.4 mA h g^−1^ at 0.2 A g^−1^ after 300 cycles). Kinetics analysis, *in situ* EIS, and *ex situ* SEM were conducted to investigate the mechanism of enhanced electrochemical performance by GN. This work demonstrates the great potentials of highly active GN on improving the lithium storage properties of Si, which could also be extended to other alloy-type anodes for LIBs.

## Conflicts of interest

There are no conflicts to declare.

## Supplementary Material

RA-013-D2RA06977F-s001
