# Supplementary material for: Integrating highly active graphite nanosheets into microspheres for enhanced lithium storage properties of silicon
Source: RSC Adv. 2023 Jan 30;13(6):4102–12. doi: 10.1039/d2ra06977f (PMC9890553; doi:10.1039/d2ra06977f)
Supplement: RA-013-D2RA06977F-s001 [file RA-013-D2RA06977F-s001.pdf]

Electronic Supplementary Material (ESI) for RSC Advances.
This journal is © The Royal Society of Chemistry 2023

Supplementary Information

Integrating highly active graphite nanosheet into microspheres for enhanced lithium storage properties of silicon

Yan Li [a], Dong Wang [a, b], Zhichao Liu [a], Xianzheng Liu [a], Jie Fu [a], Chunjie Zhang [c], Rui Zhang [a], Guangwu Wen [a, b*]*

[a] *School of Materials Science and Engineering, Shandong University of Technology, Zibo 255000, P. R. China.*

[b] *Shangdong Si-Nano Materials Technology Co. Ltd., Zibo 255000, P. R. China.*

[c] *School of Materials Science and Engineering, Harbin Institute of Technology, Harbin 150001, P. R. China.*

** E-mail correspondence: wangdong2@sdut.edu.cn, wengw@sdut.edu.cn*

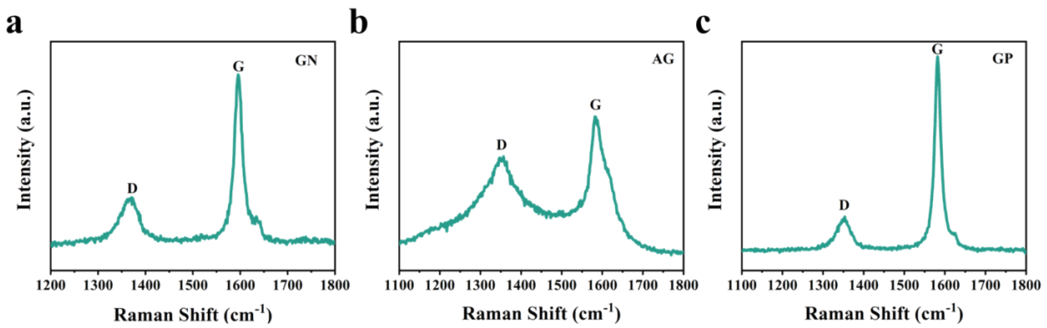

Fig. S1 Raman spectra of the (a) GN, (b) AG and (c) GP.

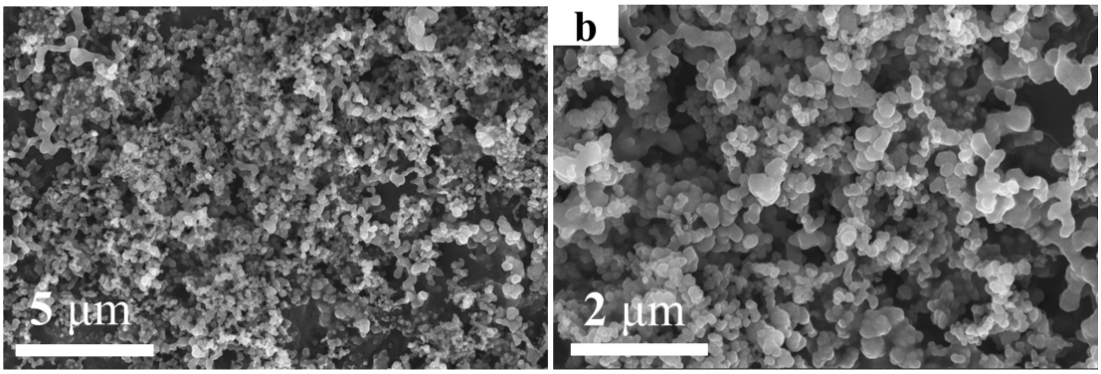

Fig. S2 SEM images of the NSi.

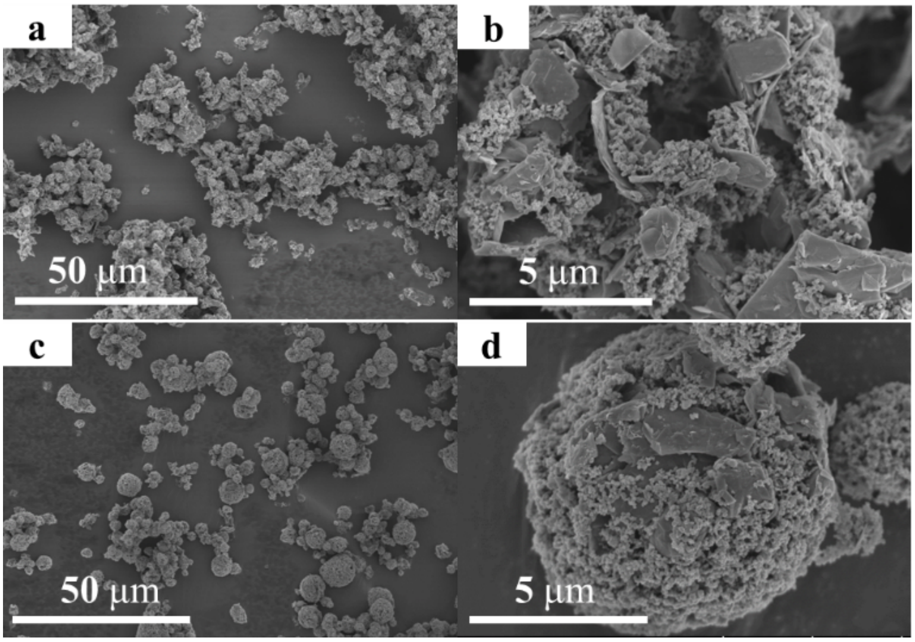

Fig. S3 SEM images of the (a, b) NSi-GN-C-0.5 and (c, d) NSi-GN-C-1.5.

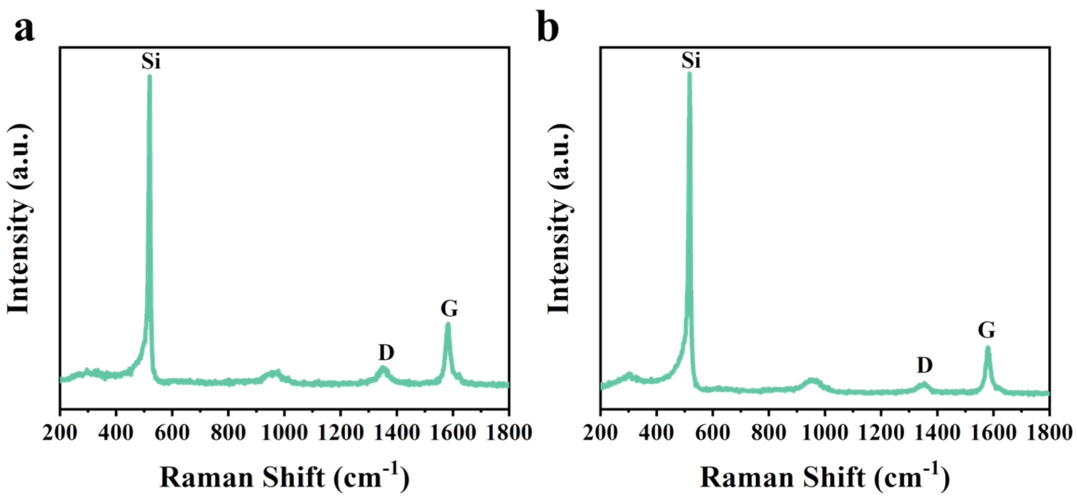

Fig. S4 Raman spectra of (a) NSi-GN-C-0.5 and (b) NSi-GN-C-1.5.

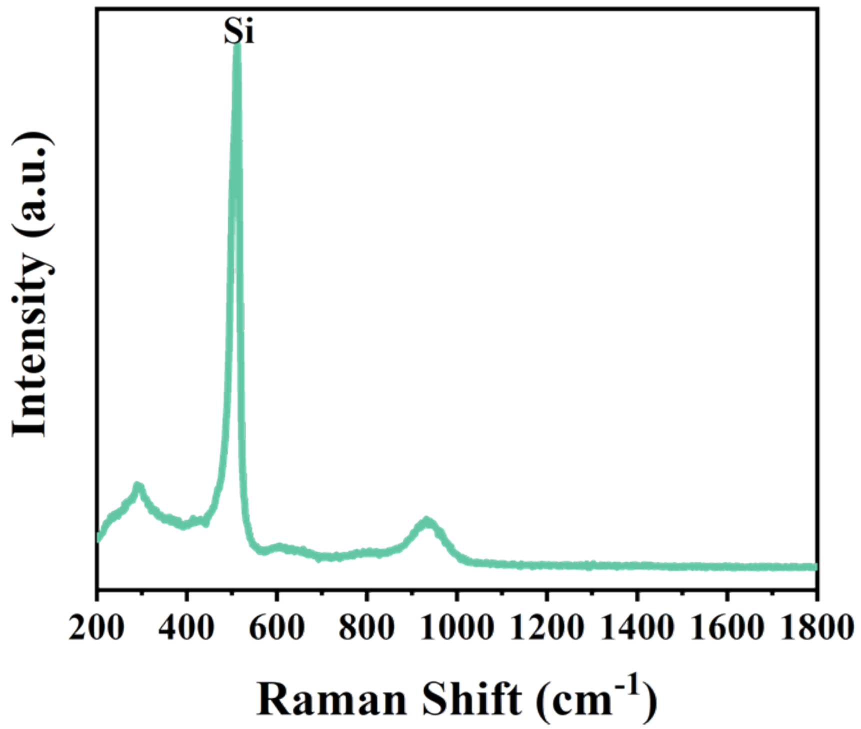

Fig. S5 Raman spectrum of the NSi.

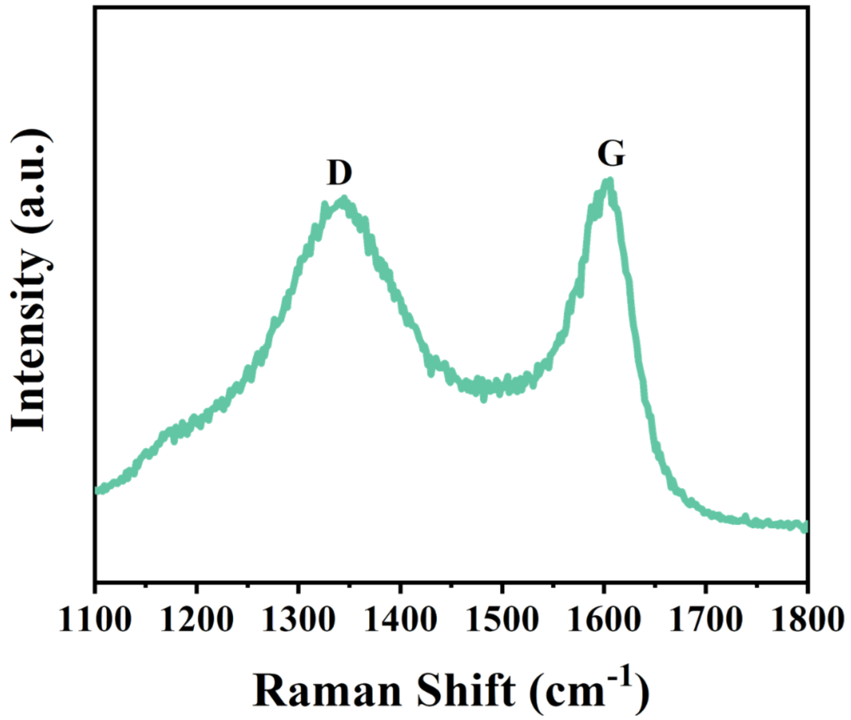

Fig. S6 Raman spectrum of the glucose-derived amorphous carbon.

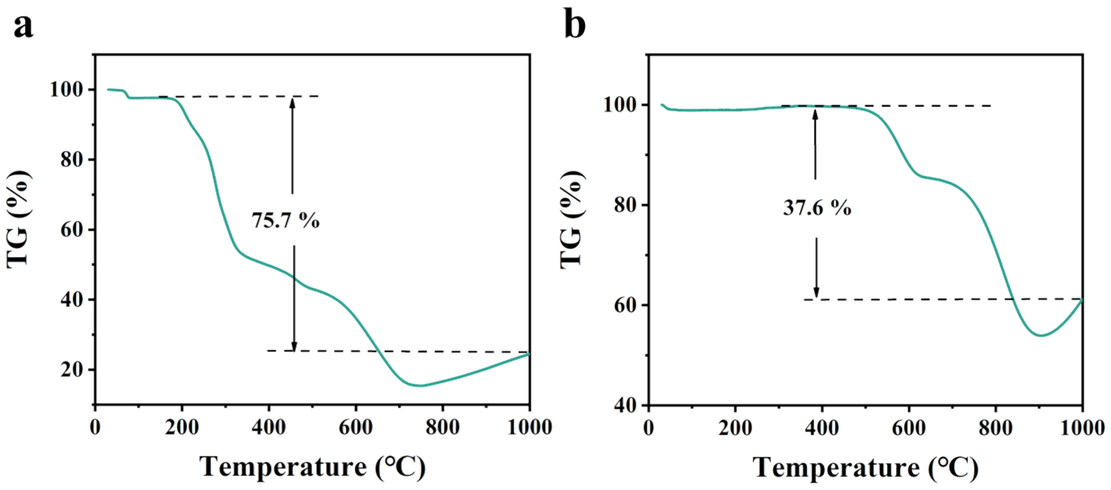

Fig. S7 TGA curves of the (a) NSi-GN-C-0.5 and (b) NSi-GN-C-1.5.

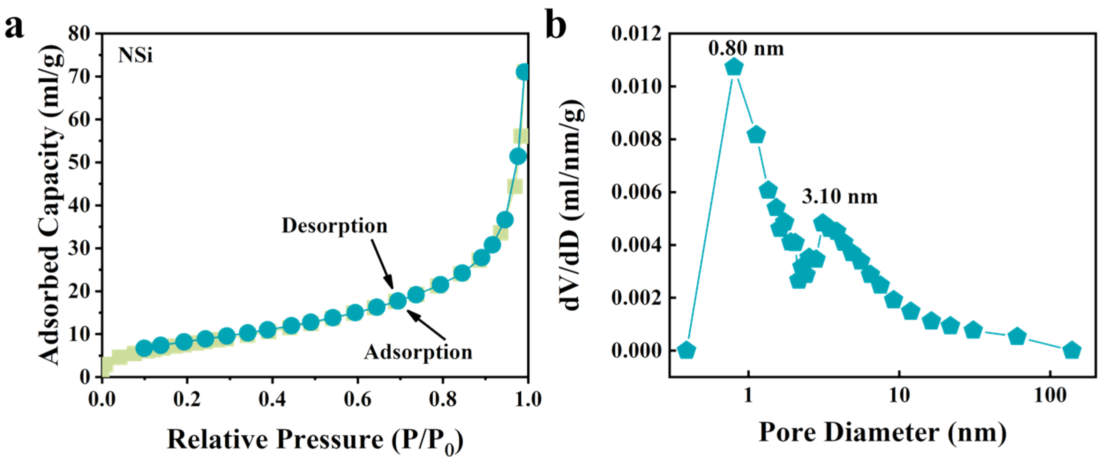

Fig. S8 (a) Nitrogen adsorption/desorption isotherms and (b) pore size distribution plot of the NSi.

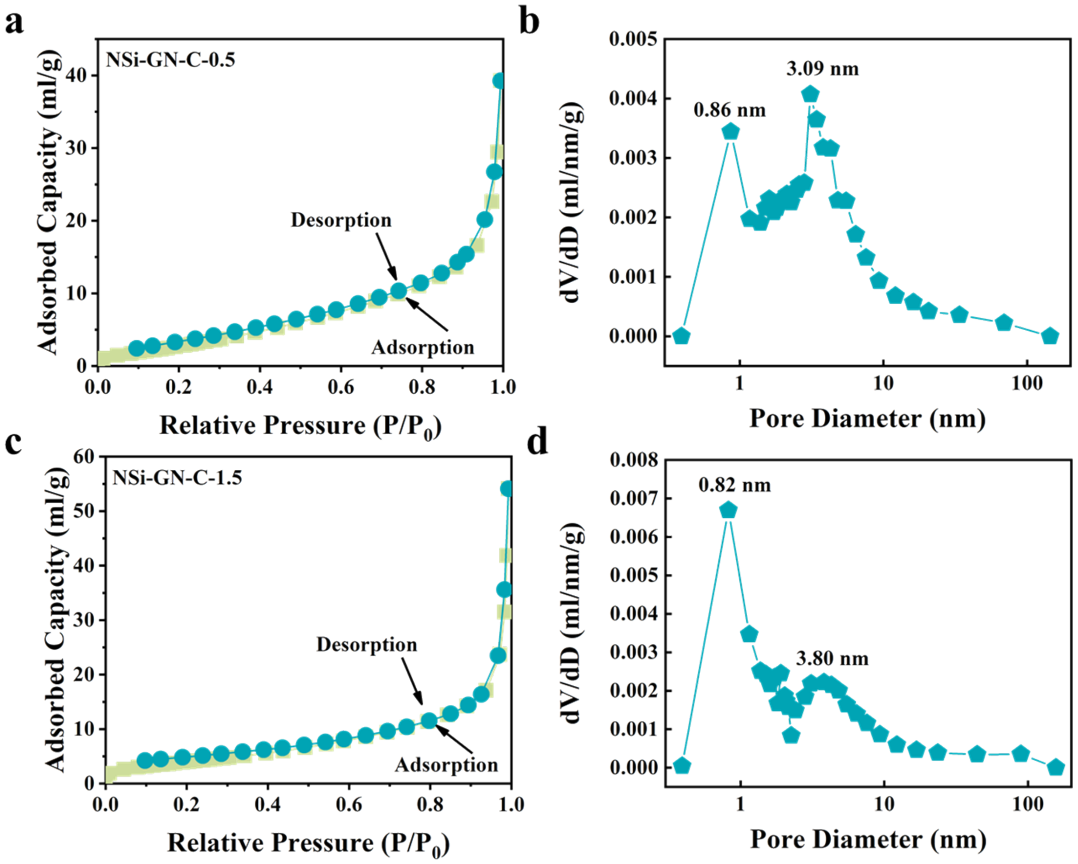

Fig. S9 Nitrogen adsorption/desorption isotherms of (a) NSi-GN-C-0.5 and (c) NSi-GN-C-1.5. Pore size distribution plots of (b) NSi-GN-C-0.5 and (d) NSi-GN-C-1.5.

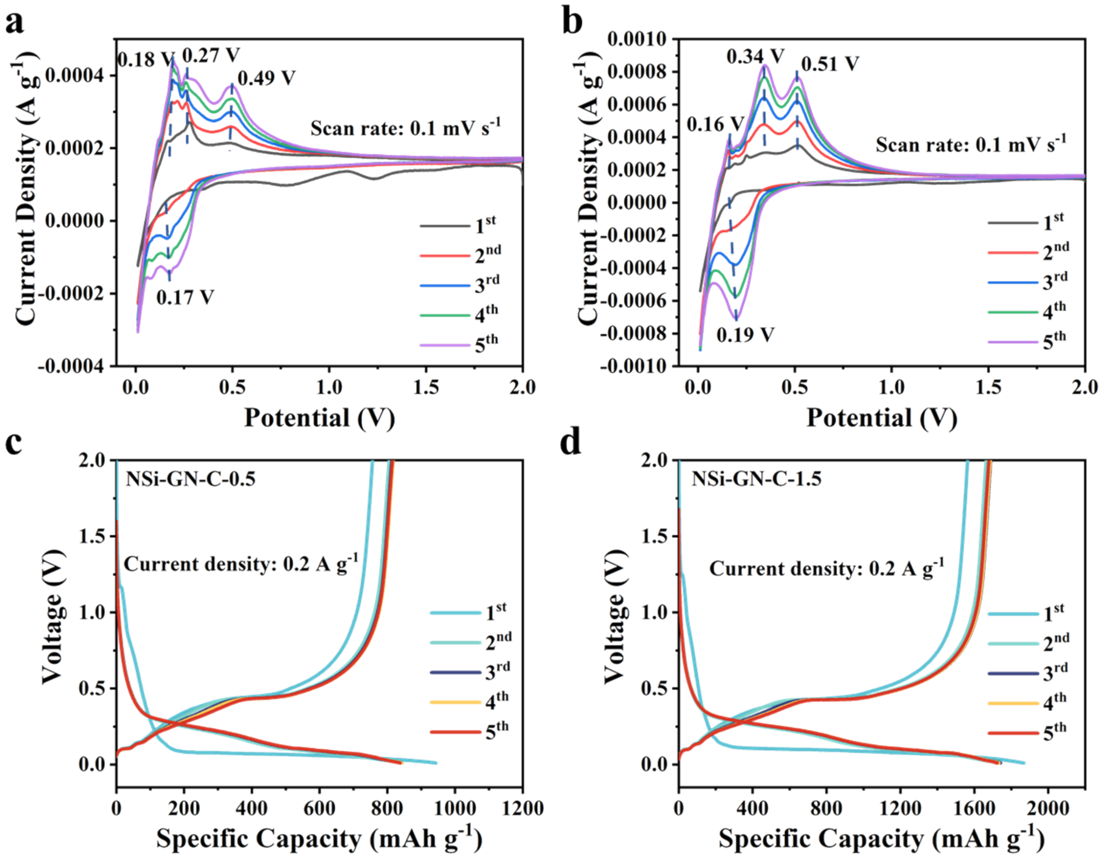

Fig. S10 CV curves of (a) NSi-GN-C-0.5 and (b) NSi-GN-C-1.5. Charge/discharge curves of (c) NSi-GN-C-0.5 and (d) NSi-GN-C-1.5 microspheres at 0.2 A g^{-1} from 0.01 to 2 V.

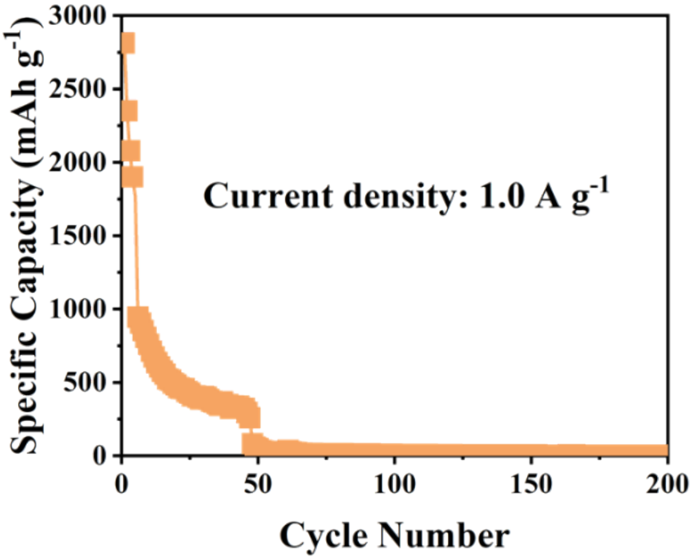

Fig. S11 Cycling performance of the NSi at 1.0 A g^{-1}.

Cyclic test of the NSi at 1.0 A g^{-1} shows that the specific capacities fade quickly.

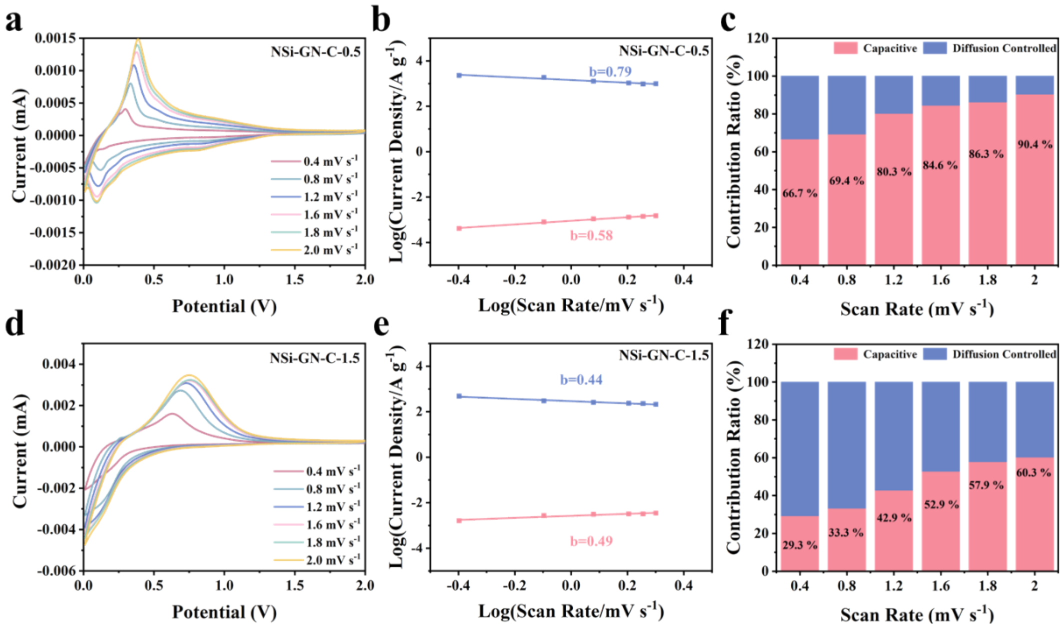

Fig. S12 (a) CV curves of NSi-GN-C-0.5 electrode at different scan rates. (b) Relationship between peak current and scan rate. (c) Ratio of capacitive and diffusion-controlled contributions at different scan rates of the NSi-GN-C-0.5. (d) CV curves of NSi-GN-C-1.5 electrode at different scan rates. (e) Relationship between peak current and scan rate. (f) Ratio of capacitive and diffusion-controlled contributions at different scan rates of the NSi-GN-C-1.5.

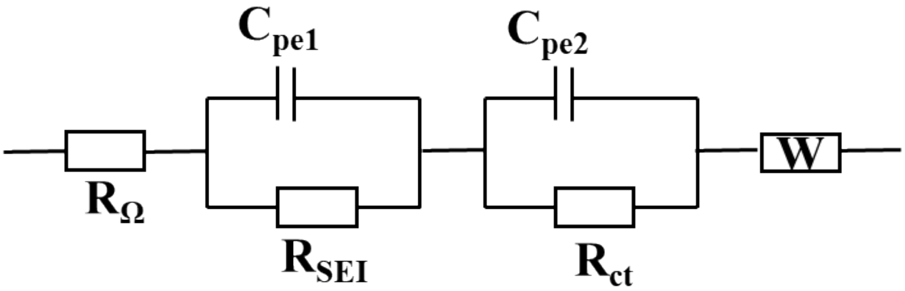

Fig. S13 Equivalent circuit corresponding to in situ impedance.

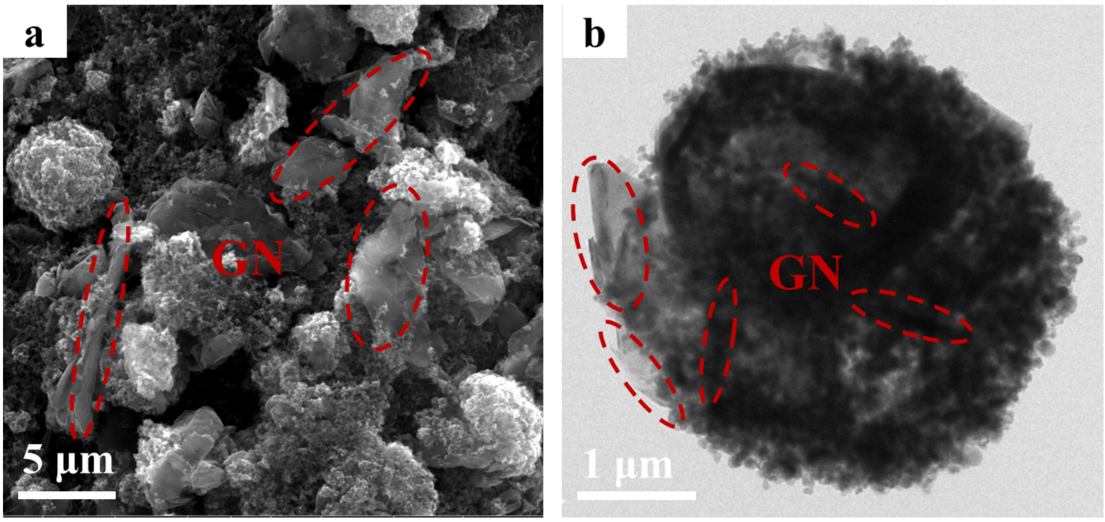

Fig. S14 (a) SEM image of the NSi-GN-C after 200 cycles. (b) TEM image of the NSi-GN-C after 200 cycles.
